# TEMPOL Enhances Polyethylene Glycol Axon Fusion Following Sciatic Nerve Transection in Adult Rats

**DOI:** 10.1021/acschemneuro.5c00677

**Published:** 2026-02-27

**Authors:** Lynn Ana Flavia Salamanca-Guillén, Liana Melo-Thomas, Kelly C. S. Roballo, André Schwambach Vieira, Bruno Henrique de Melo Lima, Luciana Politti Cartarozzi, Alexandre Leite Rodrigues de Oliveira

**Affiliations:** † Laboratory of Nerve Regeneration, Department of Structural and Functional Biology, Institute of Biology, 28132 University of Campinas, 13083-907 Campinas, São Paulo, Brazil; ‡ Department of Systemic Neuroscience, Institute of Anatomy and Cell Biology, 9377 Philipps-University of Marburg, 35037 Marburg, Germany; §Edward Via College of Osteopathic Medicine and ¶Virginia Maryland College of Veterinary Medicine, Virginia Tech, Blacksburg, Virginia 24061, United States; ∥ Department of Biochemistry and Tissue Biology, Institute of Biology, University of Campinas, 13083-862 Campinas, São Paulo, Brazil; ⊥ Center for Gender-Specific Biology and Medicine (CGBM), 13083-862 Campinas, São Paulo, Brazil

**Keywords:** nerve fusion, methylene blue, tempol, nerve repair, polyethylene glycol fusion, fusogens, functional recovery, nerve regeneration

## Abstract

Neurotmesis, the most severe form of peripheral nerve injury, involves complete transection and loss of motor and sensory function. Surgical repair, typically via end-to-end neurorrhaphy (NRR), is often required. A key pathological feature is Wallerian degeneration (WD) in the distal stump, which, along with slow axonal regeneration, leads to muscle atrophy and poor functional recovery. Polyethylene glycol (PEG)-mediated axonal fusion has emerged as a promising strategy to bypass WD by rapidly reconnecting severed axons and restoring conduction. Methylene blue (MB) has previously enhanced PEG-fusion outcomes. This study investigated whether TEMPOL (TMP), a potent antioxidant, could further improve PEG-fusion-mediated repair following sciatic nerve transection in rats. Adult female Lewis rats underwent unilateral sciatic nerve transection and were randomly assigned to one of three groups: end-to-end neurorrhaphy (NRR), MB-PEG-fusion (MB-fusion), or TEMPOL-PEG-fusion (TMP-fusion). Functional recovery was assessed for 8 weeks using CatWalk gait analysis (Peroneal Functional Index, PFI). Electrophysiological recordings (CMAPs) were obtained at baseline, immediately post-repair, and at 8 weeks. Histological and immunofluorescence analyses (neurofilament, S100, synaptophysin, GFAP, Iba-1) were performed to evaluate axonal integrity, Schwann cell activity, synaptic coverage, and glial response. TMP-fusion significantly improved motor recovery compared to MB-fusion and NRR. Animals treated with TMP-fusion demonstrated superior sensorimotor function by week 8 (PFI; *p* = 0.0232, step sequence; *p* < 0.05), enhanced nerve conduction (CMAPs amplitude, *p* < 0.05), preserved axonal morphology (NF; *p* < 0.0001, S100, *p* = 0.0004), and reduced glial activation (Iba-1, *p* = 0.0035; GFAP, *p* = 0.001) compared to NRR. Synaptic integrity in the spinal cord was better maintained in the TMP-fusion group, indicating a more complete restoration of neuromuscular connectivity (*p* < 0.0001; *p* = 0.0003). TEMPOL-PEG-fusion significantly enhances structural and functional recovery after neurotmesis, outperforming current gold-standard techniques. These results support TMP-fusion as a promising strategy for peripheral nerve repair.

## Graphical abstract

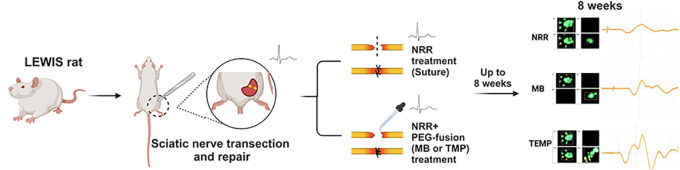

## Introduction

Peripheral nerve injuries (PNIs), resulting from transection or compression, represent a significant clinical challenge, often leading to permanent sensory loss, paralysis, and muscle weakness. PNIs affect millions of individuals worldwide annually and are observed in approximately 2–5% of all trauma cases.^1–6^ Addicionally, peripheral neuropathy— a broader category that includes PNIs— affects 2–8% of the general population, with prevalence rising to approximately 24% among older adults with peripheral neuropathy.^7–11^

The Seddon-Sunderland classification system grades PNIs based on severity, ranging from neurapraxia (Grade I), which is fully reversible, to neurotmesis (Grade V), the most severe form, involving complete nerve transection and epineural disruption. This type of injury requires surgical intervention, with end-to-end neurorrhaphy representing the gold standard approach.^12,13^ Nevertheless, nerve regeneration is slow (∼1 mm/day), and while Schwann cells play a key role in guiding axonal regrowth, incomplete remyelination and muscle atrophy often lead to persistent functional deficits.^14–16^ Conventional repair strategies— including neurorrhaphy, nerve grafts, and synthetic conduits— provide limited reinnervation and fail to prevent Wallerian degeneration.^2,13,17,18^

Interestingly, some invertebrate species are capable of restoring nerve function via axonal fusion, a phenomenon that has inspired the development of polyethylene glycol (PEG)-mediated axonal fusion protocols in mammalian models.^19,20^ Polyethylene glycol (PEG) has emerged as a promising agent for axonal fusion, capable of rapidly restoring axonal continuity and preventing Wallerian degeneration,^21–24^ achieving up to 97% motor function recovery in rodent sciatic nerve crush model, though electrophysiological recovery remains modest (25–30%).^25,26^

To enhance PEG-fusion efficacy, methylene blue (MB) has been incorporated as an antioxidant that reduces intracellular vesicles and scavenges reactive oxygen species.^25,27^ The standard protocol involves pretreatment with hypotonic saline, MB application, neurorrhaphy, PEG application, and isotonic saline rinsing. The incorporation of MB into the PEG-fusion protocol has been shown to significantly improve axonal repair and behavioral recovery, reaching 70–85% restoration at 12 weeks postinjury.^14^ Due to cytotoxic effects at higher concentrations, clinical formulations are limited to 0.5% to ensure safety while maintaining efficacy.^28^

TEMPOL (TMP), a water-soluble antioxidant that mimics superoxide dismutase, reduces oxidative stress, apoptosis, and axonal damage while enhancing motor recovery and synaptic preservation in PNIs.^29–37^ Despite its neuroprotective potential, Tempol has only been administered systemically (e.g., oral or intraperitoneal routes) and has not yet been integrated into PEG-fusion protocols for nerve repair. Its promising properties warrant further investigation for enhancing functional recovery in PNIs. Therefore, the present study investigated the PEG-fusion effectiveness comparing Tempol against MB following sciatic nerve end-to-end neurorrhaphy. The injured tissue repair was monitored by immunohistochemistry, and the sciatic nerve functional recovery was followed with the walking track test and electroneuromyography.

## Results and Discussion

### Functional Recovery (Figure 1)

Motor function was assessed using the CatWalk system by analyzing three parameters: Peroneal Functional Index (PFI), Regularity Index, and Base of Support. Regarding PFI, the TMP-fusion group exhibited the most robust recovery at 8 weeks postsurgery, showing significant improvement compared to the NRR group (*p* = 0.0232). Significant increases were detected at weeks 2, 3, 4, 5, and 8 (*p* = 0.0271, *p* = 0.0043, *p* = 0.0380, *p* = 0.0003, and *p* = 0.0232, respectively), whereas the MB-fusion group differed significantly from NRR only at week 1. The NRR group showed no functional recovery throughout the evaluation period. Transient fluctuations in PFI values during midregeneration suggest possible paresthesia, a phenomenon commonly associated with active reinnervation (Figure 1A).

**Figure 1:**
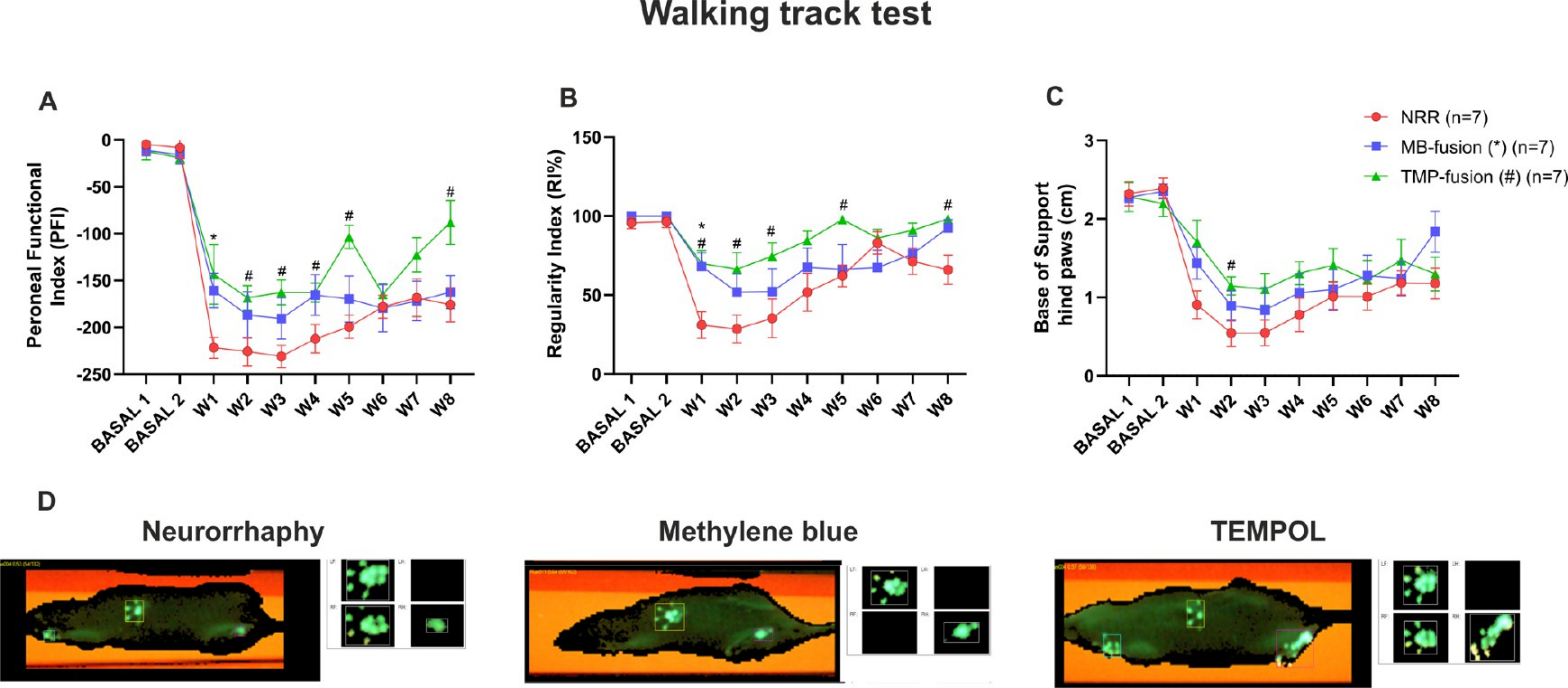
Sensorimotor recovery over 8 weeks. CatWalk analysis was performed weekly using three parameters: Peroneal Functional Index (PFI), Regularity index (%), and Base of Support (BOS, cm). (A) PFI at 8 weeks shows a significant improvement in the TMP-fusion group vs NRR. (B) The regularity index indicates superior recovery in the TMP-fusion group across the 8-week period. (C) BOS analysis revealed few significant differences between PEG-fusion groups and control. (D) Representative images from the Walking Track Test evidencing the greater functional recovery of Tempol-PEG-fusion at the end of the 8-week period, followed by the MB-PEG-fusion treatment, while the control group showed the least recovery, as evidenced by the pawprint pattern. Data include two baseline measures (BASAL 1, BASAL 2) and weekly intervals (W1–W8). Statistical comparisons: NRR vs MB-fusion (*); TMP-fusion (#); *p* < 0.05, Two-way ANOVA.

For the Regularity Index, the TMP-fusion group again demonstrated the strongest improvement, with significantly higher values at weeks 1, 2, 3, 5, and 8 (*p* = 0.0112, *p* = 0.0320, *p* = 0.0433, *p* = 0.0029, and *p* = 0.0227, respectively) compared to NRR. The MB-fusion group showed a significant increase only at week 1 (*p* = 0.0179) (Figure 1B). Notably, both PEG-fusion groups (MB and TMP) performed better than NRR during the initial 4 weeks. By week 8, both fusion groups reached values approximating normal locomotor performance (RI ≈ 100), whereas the NRR group displayed minimal improvement.

The Base of Support parameter was better preserved in the TMP-fusion group at week 2 (*p* = 0.0287), although overall intergroup differences were less pronounced (Figure 1C).

Overall, Tempol-PEG-fusion treatment produced superior recovery of motor function and gait coordination by week 8, with signs of early functional restoration observed during the first week postinjury, consistent with a successful axonal fusion process.

The sham-operated animals exhibited stable locomotor performance throughout the four-week evaluation period. Neither PFI, Regularity Index, nor Base of Support showed significant variation over time (Supporting Figure S1A–C). Gait-cycle images and footprint patterns (Supporting Figure S1D) demonstrated symmetrical stepping and consistent paw placement across sessions, confirming preserved motor coordination and the absence of gait alterations. This stability reinforces that sciatic nerve surgical exposure alone does not interfere with motor performance.

Complete transection of a peripheral nerve causes severe deficits, including sensory loss, impaired muscle contraction, and loss of voluntary movement.^26^ These outcomes result from Wallerian degeneration—a progressive breakdown of myelin, axons, and neuromuscular junctions (NMJs) —which begins within hours of injury and can persist for months, often leading to irreversible functional loss.^38^ This is primarily due to the slow rate of endogenous axonal regeneration (∼1 mm/day), which cannot outpace the rapid muscle atrophy that follows denervation. Consequently, despite various surgical techniques, especially in proximal injuries, functional recovery remains limited—even with the gold-standard neurorrhaphy.^2,28,39–42^

PEG-fusion has emerged as a promising alternative, combining a fusogen (PEG), antioxidant agent (MB), and calcium-modulating solutions to facilitate immediate axonal continuity and prevent degeneration, thereby promoting functional and structural recovery.^14,26,43^

Evaluating voluntary behavioral recovery is critical for assessing the effectiveness of peripheral nerve repair strategies. In this study, functional outcomes were monitored over an 8-week period using the walking track (CatWalk) test, focusing on the peroneal functional index (PFI), regularity index (RI), and base of support (BOS). This evaluation window aligns with previous reports demonstrating that behavioral restoration typically occurs between 1 and 6 weeks postinjury, depending on the model and intervention used.^14,22,39,43–45^ Our results show that TMP-fusion produced a significant improvement in motor performance by week 8, whereas MB-fusion did not differ significantly from the nonfused injured control. The superior performance of the TMP-fusion group is likely related to Tempol’s previously described neuroprotective actions—particularly its antioxidative and antiapoptotic effects that reduce reactive oxygen species (ROS) and mitigate secondary injury. By limiting oxidative stress, Tempol may help maintain axonal integrity and create a more permissive environment for rapid axonal reconnection and functional restoration.

Interestingly, we observed greater variability in the PFI of the TMP-fusion group during weeks 5–6, which could reflect episodes of transient paresthesia. Previous studies have reported that such fluctuations in gait parameters often accompany active reinnervation phases, during which mixed or unstable motor unit recruitment can temporarily disrupt locomotor coordination.^2,40,46–49^ This interpretation is consistent with the progressive stabilization observed at later time points.

As expected, sham-operated animals showed no significant changes over time in any behavioral parameter. Their gait patterns remained comparable to those of uninjured animals throughout the study. This finding validates the use of the sham group as a reliable baseline control and confirms that sciatic nerve surgical exposure alone did not alter motor performance. Therefore, the functional deficits observed in the injured groups can be attributed specifically to the sciatic nerve transection and not to nonspecific manipulation during surgery.

### Electrophysiological Recovery

Electroneuromyography (ENMG) was performed at three time points: before injury, immediately after treatment, and 8 weeks postinjury. Key parameters—including latency, total duration, positive duration, total amplitude, and positive amplitude of compound muscle action potentials (CMAPs)—were measured to assess electrical conduction (Figure 2). No significant differences were observed among groups before injury, confirming equivalent baseline electrophysiological function. As expected, CMAPs were not detected in the NRR group immediately after treatment, resulting in nearly zero values for all parameters at this time point, reflecting complete loss of axonal continuity following transection.

**Figure 2:**
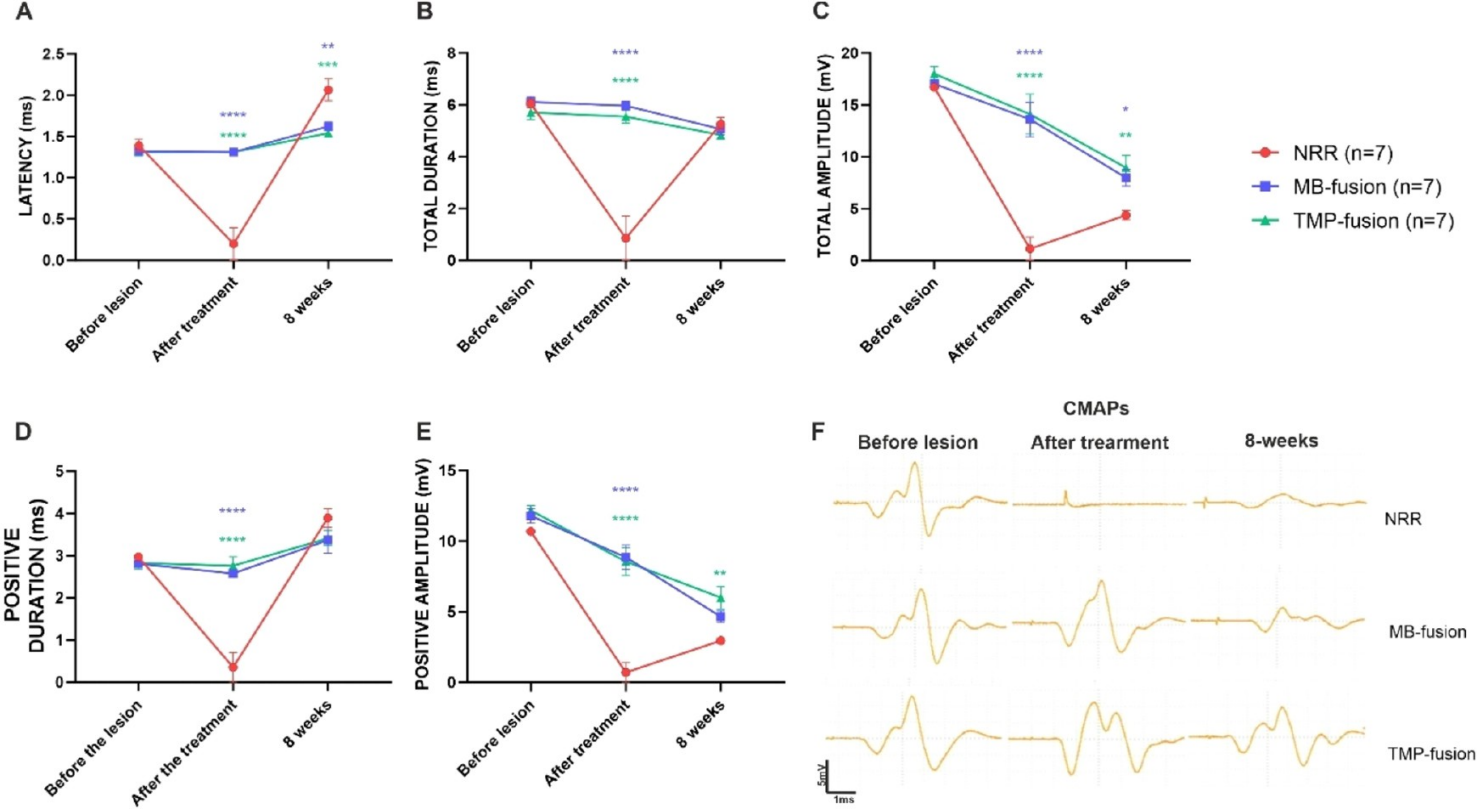
Electrophysiological recovery. Electroneuromyography was used to record the compound muscle action potential (CMAP) of the cranial tibial muscle after stimulation proximal to the surgical repair. (A) latency, (B) total duration, (C) total amplitude, (D) positive duration and (E) positive amplitude of the CMAPs were compared between groups: Neurorraphy (NRR), MB-PEG fusion (MB-fusion) and Tempol-PEG-fusion (TMP-fusion). One-way ANOVA was used for each time point, *p* < 0.05­(*), *p* < 0.005­(**), *p* < 0.0005­(***), *p* < 0.0001­(****). CMAPs of the different groups in three time points: before the lesion, immediately after the treatment and after 8 weeks postsurgery. (F) Representative CMAP recordings illustrating the functional outcomes. The NRR group showed the greatest impairment, while TMP-fusion (TMP) yielded the best recovery, including greater CMAP amplitudes. CMAPs were recorded at baseline, post-treatment, and at 8 weeks; scale: 1 ms (time), 5 mV (amplitude).

All PEG-fusion treatments demonstrated significant improvements in latency (Figure 2A), duration (Figure 2B,C), and amplitude (Figure 2D,E) immediately after treatment compared to the NRR group (*p* < 0.0001). These findings reflect the absence of CMAPs in the NRR group. At 8 weeks postinjury, the control group exhibited significantly higher latency compared to all PEG-fusion treatments (*p* < 0.05). Although all groups presented increased latency relative to preinjury levels, PEG-fusion groups displayed values that remained closer to baseline, indicating superior restoration of conduction velocity (Figure 2A).

No significant differences between groups were observed in total duration (Figure 2B) or positive duration (Figure 2C) of CMAPs at 8 weeks; however, PEG-fusion groups—particularly TMP-fusion—showed significant recovery of total (Figure 2D) and positive amplitude (Figure 2E) (*p* < 0.05), supporting improved reinnervation and neuromuscular transmission. Overall, all PEG-fusion treatments, particularly TMP-fusion, effectively reduced electrical conductivity delay and restored CMAPs immediately after treatment, producing higher amplitudes compared to NRR treatment.

CMAP traces recorded at all three time points (Figure 2F) illustrate that PEG-fusion induced immediate CMAP recovery, with waveforms closely resembling preinjury profiles, while the control group exhibited no detectable responses immediately after treatment. By 8 weeks, CMAPs remained present in all PEG-fusion groups, though amplitudes were slightly lower than immediately post-treatment, with TMP-fusion showing the highest degree of recovery.

The sham group exhibited intact electrophysiological conduction, as evidenced by intraoperative ENMG recordings that showed robust CMAPs with normal amplitude and duration (Supporting Figure S1E). The stability of these parameters confirms that the surgical exposure procedure alone does not alter nerve excitability or signal propagation. This electrophysiological consistency provides a reliable reference point for distinguishing true injury-related deficits and for interpreting the recovery achieved by the experimental treatments.

Tempol treatment promoted superior early electrophysiological recovery within the first week postinjury, indicating that effective axonal fusion occurred shortly after repair. Across the full 8-week period, TMP-fusion consistently produced better outcomes—shorter latencies and higher CMAP amplitudes—than NRR. These improvements likely arise because Tempol reduces myelin disruption and limits fibrotic tissue formation, both of which are pathological features known to hinder axonal regeneration and restrict functional recovery. By preserving myelin integrity and minimizing the development of collagen-rich barriers, Tempol creates a more permissive environment for sustained electrical conduction and axonal survival.^29,50–60^

Peripheral nerve injury triggers inflammation and bleeding, activating fibroblasts and stromal cells to produce collagen and form fibrotic barriers. In severe injuries such as neurotmesis, this matrix inhibits axonal regrowth by creating both physical and biochemical obstacles.^61–65^ Notably, animals treated with TMP- or MB-fusion showed signs of attenuated fibrosis, as inferred from improved nerve conduction. This suggests that both antioxidants may enhance the regenerative environment by mitigating oxidative stress and modulating fibroblast activation.

Both treatments appear to reduce demyelination and fibrosis, with TMP-fusion leading to faster and more sustained electrophysiological recovery. These effects are consistent with prior reports on MB, which stabilizes membranes, prevents axonal collapse, and maintains open axonal ends by reducing extracellular vesicle formation.^66,67^ MB also scavenges ROS, limits oxidative damage, and promotes neuronal survival by inhibiting caspase activation.^68–70^ Similarly, TEMPOL neutralizes free radicals,^71,72^ decreases vesicle formation, and preserves myelin by inhibiting lipid peroxidation in Schwann cells and oligodendrocytes.^73,74^ These antioxidant properties likely contribute to the enhanced efficacy of PEG-fusion,^14,39,75^ as supported by immunofluorescence data showing greater neurofilament and S100 preservation in TMP- and MB-fusion groups—indicators of improved axonal integrity and Schwann cell support.

The potent electrophysiological effects of Tempol suggest stronger neuroprotective activity compared to methylene blue (MB). Although both fusion groups exhibited reduced CMAPs, this may stem from mechanical disruption of fused axons due to natural limb movement, underscoring the need for protective devices to enhance nerve stability. Additionally, nonspecific or unstable fusions may degenerate, impairing conduction. Our data suggest that Tempol may reduce extracellular vesicle formation more effectively than MB, improving PEG-fusion success and PEG-seal formation. Its pronounced antioxidant and antiapoptotic properties likely mitigate neuroinflammation, preserve myelin, and maintain axonal conduction—thus more effectively inhibiting Wallerian degeneration. Morphological analysis further supports this, showing greater axonal preservation in TMP-fusion compared to MB and NRR, indicating Tempol not only protects fused axons but may also enhance survival of nearby nonfused fibers.^26,67^

In the sham group, electrophysiological recordings remained stable throughout the evaluation period. Intraoperative ENMG demonstrated normal CMAPs with physiological amplitude and duration, confirming that sciatic nerve exposure and manipulation alone did not impair electrical conduction. This preserved electrophysiological profile validates the sham group as a reliable baseline control and reinforces that all conduction deficits observed in experimental groups were attributable to the nerve transection rather than nonspecific surgical effects.

Electrophysiological analysis of the electron microscopy cohort at 2 weeks further supports the advantages of PEG-fusion. In these animals (*n* = 3 per group), CMAPs remained absent in the NRR condition, consistent with ongoing Wallerian degeneration and lack of early reconnection. In contrast, both MB- and TMP-fusion groups displayed measurable CMAPs at the 2-week time point. TMP-fusion showed higher amplitudes and shorter latencies, indicating better maintenance of conduction across the repair site. These electrophysiological findings parallel the morphological observations obtained distal to the fusion site. (see Supporting Figures S2).

### Immunofluorescence

#### Tempol Enhances Axonal Preservation

Axonal preservation was assessed via antineurofilament immunolabeling in three treatment groups: NRR, MB-fusion, and TMP-fusion, at 8 weeks postsurgery. Quantification was performed by calculating the integrated density of pixels ratio between ipsilateral and contralateral nerves at both proximal and distal extremities. Image analysis was conducted using IMAGEJ software with enhanced contrast and thresholding tools at 200× magnification (Figure 3A–D).

**Figure 3:**
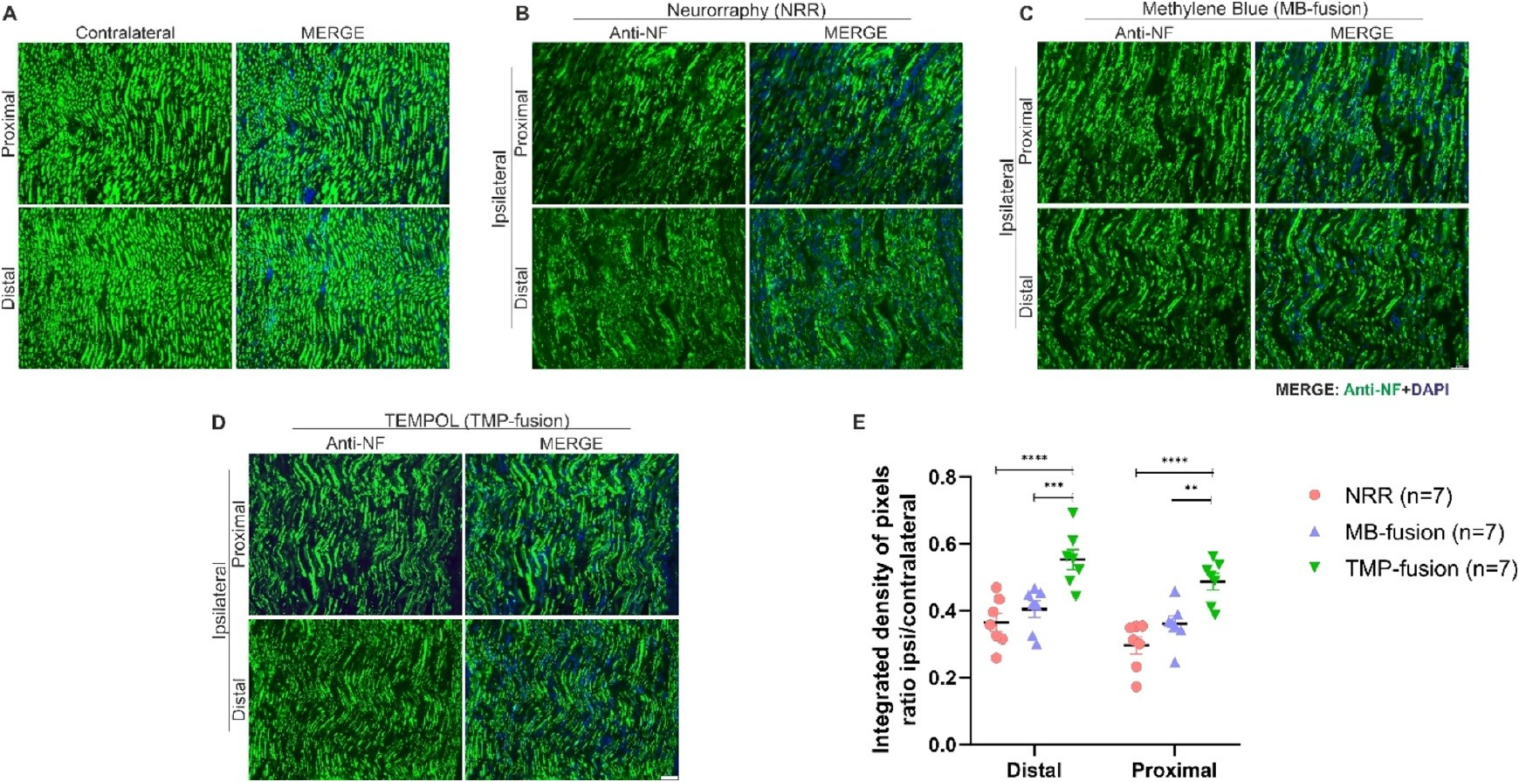
Morphological preservation. Rats were euthanized, and nerve samples were analyzed by immunohistochemistry for neurofilaments at proximal and distal sites relative to the lesion. Representative images of neurofilament immunolabeling are shown for: (A) contralateral nerve, (B) neurorrhaphy (NRR), (C) MB-fusion, and (D) TMP-fusion. TMP-fusion treatment demonstrates significant preservation of the nerve microenvironment, correlating with improved functional recovery. (E) Quantification of neurofilament immunoreactivity. Two-way ANOVA: *p* < 0.05 (*), *p* < 0.01 (**), *p* < 0.001 (***), *p* < 0.0001 (****). Scale bar: 50 μm.

Quantitative analysis showed greater immunoreactivity and axonal preservation in the distal extremity than in the proximal extremity across all treatments, with the distal region more closely resembling the contralateral nerve. Among treatments, TMP-fusion exhibited significantly greater preservation than NRR (*p* < 0.0001). In the proximal extremity, TMP-fusion showed significantly higher immunoreactivity compared to MB-fusion (*p* = 0.0049) and NRR (*p* < 0.0001). Similarly, in the distal extremity, TMP-fusion demonstrated significantly greater immunoreactivity than MB-fusion (*p* = 0.0009) and NRR (*p* < 0.0001), while no significant differences were found between MB-fusion and NRR (*p* = 0.542) (Figure 3E).

In summary, these results indicate that TMP-fusion enhances axonal morphology preservation in both proximal and distal extremities of the distal stump, with a more pronounced effect in the distal region, closer to the injury and repair site.

Our data further show that Tempol treatment leads to superior axonal morphology preservation compared to both MB and NRR, in regions proximal and distal to the lesion. This enhanced protection likely results from Tempol’s potent neuroprotective effects, which—combined with PEG-fusion—promote survival of fused axons and support adjacent nonfused segments,^26,67^ effectively reducing Wallerian degeneration. Notably, greater preservation in the distal stump reinforces the idea that Tempol more effectively rescues distal nerve integrity than MB or NRR, consistent with behavioral and electrophysiological outcomes.

The observed reduction in fibrotic scarring supports our hypothesis that Tempol modulates fibrotic responses at the injury site but we need more specific evidence yet. These results align with previous studies reporting that antioxidant treatments regulate Schwann cell activation and promote a regenerative microenvironment conducive to nerve repair.^76–78^

#### Schwann Cells Behavior after Fusion

Schwann cell presence was analyzed 8 weeks postinjury using the anti-S100 marker. Images of both the distal (DE) and proximal (PE) extremities were captured at 200× magnification, and quantification was performed using IMAGEJ software with enhanced contrast and density-slicing features. The percentage ratio of integrated density of pixels (ipsilateral/contralateral) was calculated separately for the proximal and distal ends of the distal stump.

Representative images indicate that the TMP-fusion treatment exhibits a closer morphological resemblance to a healthy nerve compared to the other treatments, with this effect more pronounced at the distal end than at the proximal end. The MB-fusion treatment follows, while the NRR treatment shows the least morphological preservation. All treatments displayed greater immunoreactivity in the ipsilateral nerve sections compared to the contralateral sections (Figure 4A–D).

**Figure 4:**
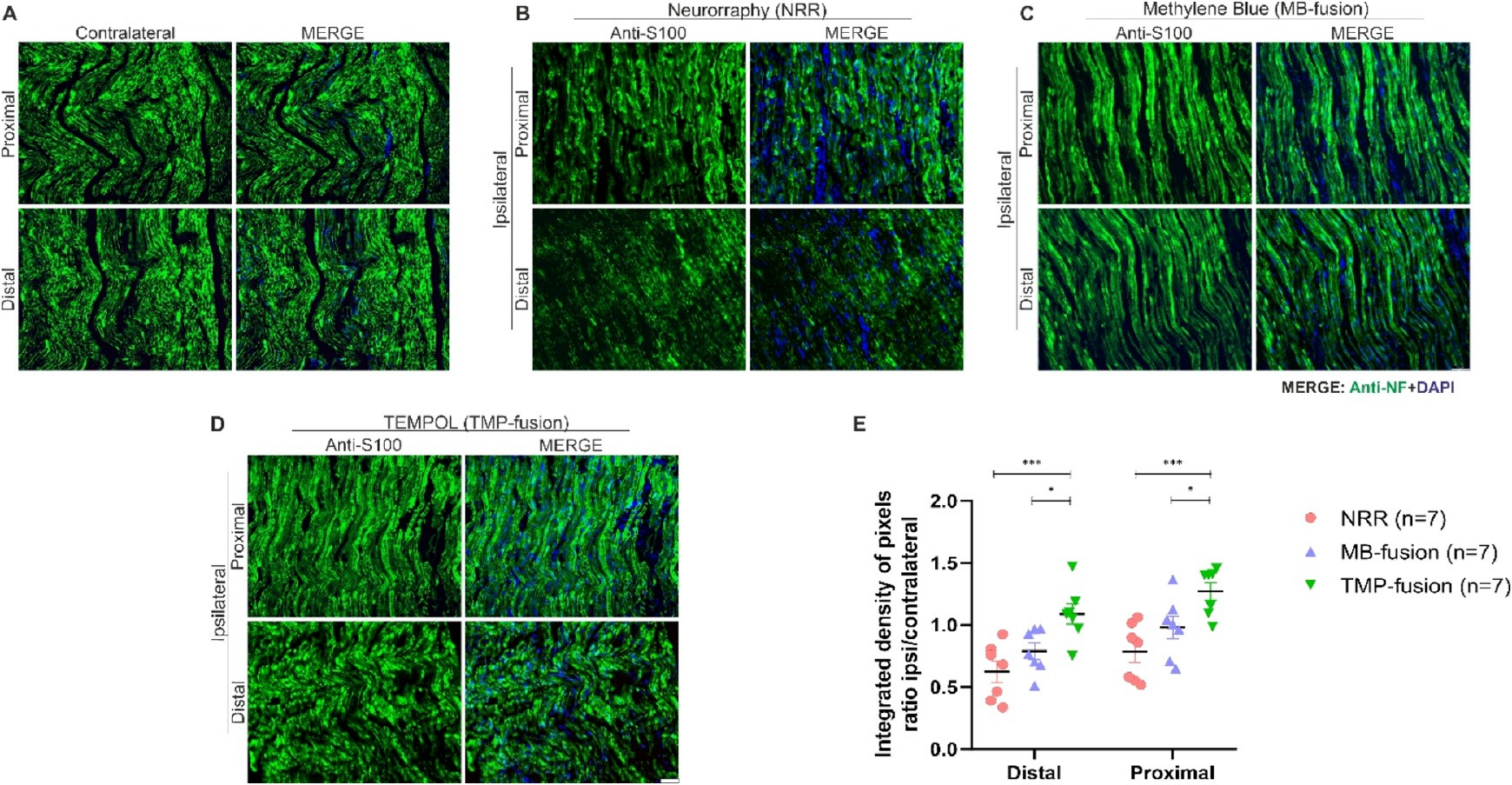
Schwann cell preservation. Ipsilateral nerves were analyzed by immunohistochemistry for S-100 to assess Schwann cell presence in proximal and distal regions relative to the lesion site. Representative images of S-100 immunolabeling are shown for: (A) contralateral nerve, (B) neurorrhaphy (NRR), (C) MB-fusion, and (D) TMP-fusion. TMP-fusion treatment resulted in a significant increase in Schwann cell presence. (E) Quantification of S-100 immunoreactivity. Statistical analysis was performed using two-way ANOVA *p* < 0.05 (*), *p* < 0.001 (**). Scale bar: 50 μm.

Quantitative analysis revealed that in the distal extremity, TMP-fusion exhibited significantly higher immunoreactivity than both MB-fusion (*p* = 0.0344) and NRR (*p* = 0.0007). Similarly, in the proximal extremity, TMP-fusion displayed higher immunoreactivity compared to both MB-fusion (*p* = 0.0402) and NRR (*p* = 0.0004). The proximal extremity demonstrated greater expression compared to the distal extremity (Figure 4E). No significant differences were observed between the MB-fusion and NRR treatments.

In summary, TMP-fusion treatment promotes increased Schwann cell activation in the injured nerve after 8 weeks, with a more pronounced expression in the proximal extremity.

Schwann cells play a central role in peripheral nerve regeneration by rapidly upregulating neurotrophic factors (NGF, BDNF, GDNF) and matrix proteins (laminin, fibronectin, collagen) that promote axonal outgrowth and form Büngner bands to guide regenerating axons.^79–82^ In our study, Tempol-PEG-fusion significantly increased Schwann cell reactivity, especially proximal to the lesion, suggesting enhanced axonal regrowth and remyelination.^83^ Elevated Schwann cell density has also been linked to reduced fibroblast activation and diminished fibrotic scarring, consistent with our findings.^84–87^ This supports the hypothesis that Tempol modulates the injury microenvironment to favor regeneration, likely by mitigating fibrotic processes. Moreover, these results align with prior in vitro studies showing that delayed repair leads to lower Schwann cell density and increased fibrosis, underscoring the relevance of antioxidants in maintaining Schwann cell function and promoting a permissive regenerative niche.^79,88^

#### Synapse Preservation

Immunostaining for synaptophysin revealed significant reduction in synaptic preservation in the lumbar enlargement following neurotmesis, with decreased synaptic density on the ipsilateral side compared to the contralateral side across all groups. This reduction was particularly noticeable in the synaptic coverage of motoneurons (Figure 5).

**Figure 5:**
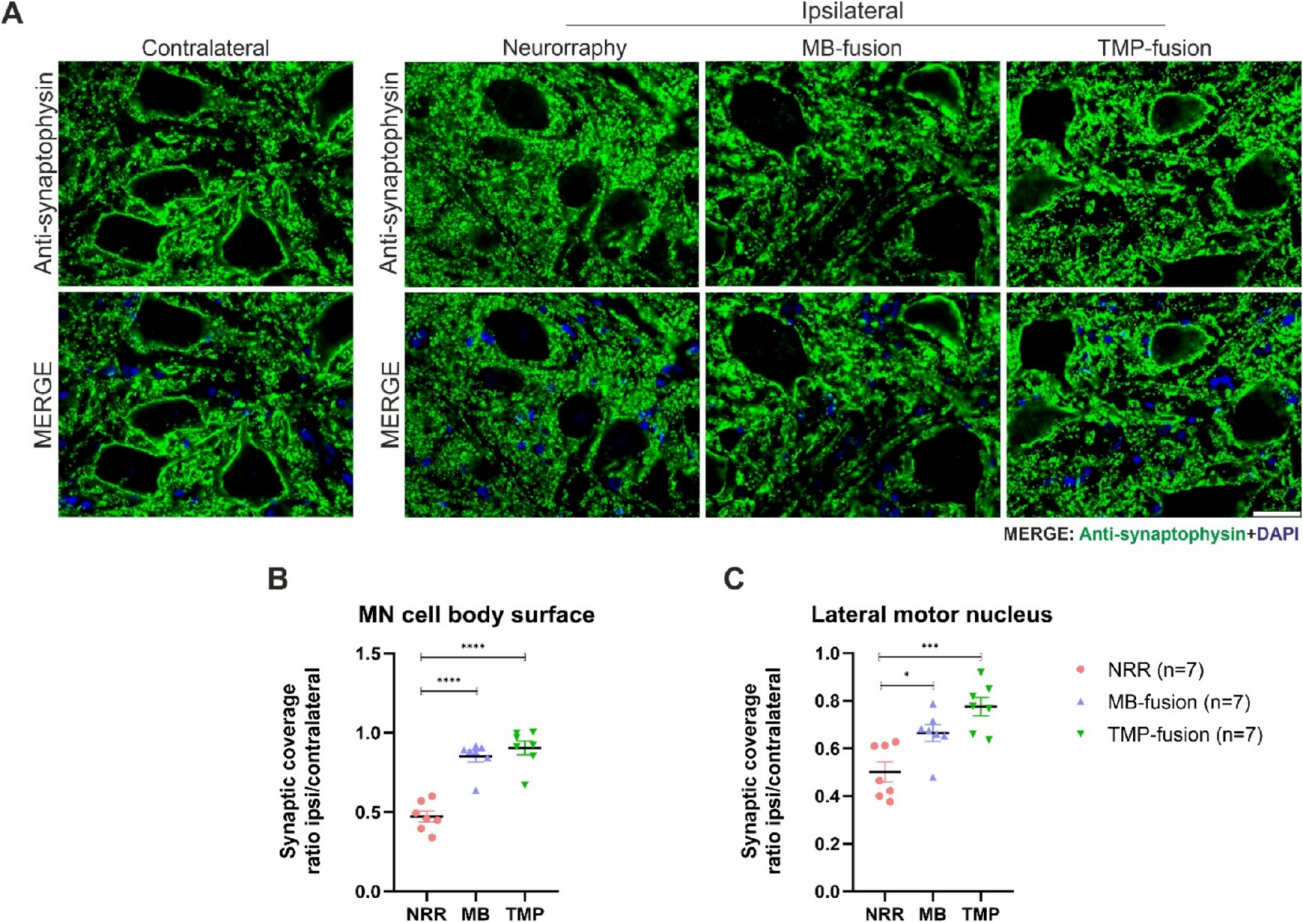
Synaptic Preservation Analysis. (A) Representative images of synaptophysin immunolabeling in the different experimental groups on the ipsilateral and contralateral sides, 8 weeks after repair (neurorrhaphy or PEG-fusion). The TMP-fusion treatment shows a significant improvement in synaptic coverage compared to the NRR group. (B, C) Quantification of synaptic coverage on the motoneuron cell body surface and across the lateral motor nucleus. One-way ANOVA with Tukey’s post hoc test was used for statistical analysis. (*p* < 0.05 (*); *p* < 0.01 (**); *p* < 0.001 (***); *p* < 0.0001 (****)). Scale bar: 50 μm.

The TMP-fusion group exhibited the highest synaptic preservation on the ipsilateral side compared to the control (Figure 5A), while the MB-fusion treatment also demonstrated significant synaptic retention relative to the control (Figure 5A). In the NRR group, synaptic loss was evident both around motoneuron (MN) cell bodies and across the entire lateral motor nucleus, with more pronounced decrease surrounding MNs.

The TMP-fusion treatment preserved synaptic coverage at 0.9 ± 0.04 (*p* < 0.0001) around motoneurons and 0.77 ± 0.04 (*p* = 0.0003) across the lateral motor nucleus. The MB-fusion group maintained synaptic coverage at 0.85 ± 0.04 (*p* < 0.0001) around motoneurons and 0.66 ± 0.04 (*p* = 0.0207) across the lateral motor nucleus (Figure 5B,C).

Synaptic preservation analysis revealed that local Tempol treatment effectively maintained spinal cord connectivity after neurotmesis. Tempol-treated animals exhibited significantly greater preservation of presynaptic terminals and synaptic coverage around lumbar motoneurons, in contrast to the NRR group, which showed marked synaptic loss. These results suggest that Tempol not only protects peripheral axons but also contributes to central synaptic integrity—likely through its antioxidant action that reduces lipid peroxidation and modulates retrograde degenerative responses.^89–93^ Normally, synaptic inputs form dense clusters, but injury disrupts this pattern, leading to decreased coverage.^94^ In our study, Tempol preserved synaptic structure and functional contacts, consistent with our previous findings demonstrating its efficacy in maintaining glutamatergic and motoneuronal inputs following spinal root and peripheral nerve lesions in neonatal models.^95,96^

#### Tempol Reduces Glial Reactivity

We investigated whether PEG-fusion treatments could modulate microglial and astrocytic reactivity associated with inflammatory processes. Immunostaining for Iba-1 and GFAP was used to assess glial reactivity in lamina IX of Rexed in the spinal cord. A significant increase in microglial and astrocytic reactivity was observed on the ipsilateral side, with the highest levels detected in the neurorrhaphy (NRR) group (Figure 6A–B). Importantly, the TMP-fusion treatment significantly reduced glial reactivity compared to the NRR treatment (*p* < 0.0001). The MB-fusion treatment also showed a significant effect on glial reactivity, with a more pronounced downregulation of astroglial GFAP (*p* < 0.001). Additionally, the microglial response was significantly reduced compared to the control group (*p* < 0.01) (Figure 6C–D).

**Figure 6:**
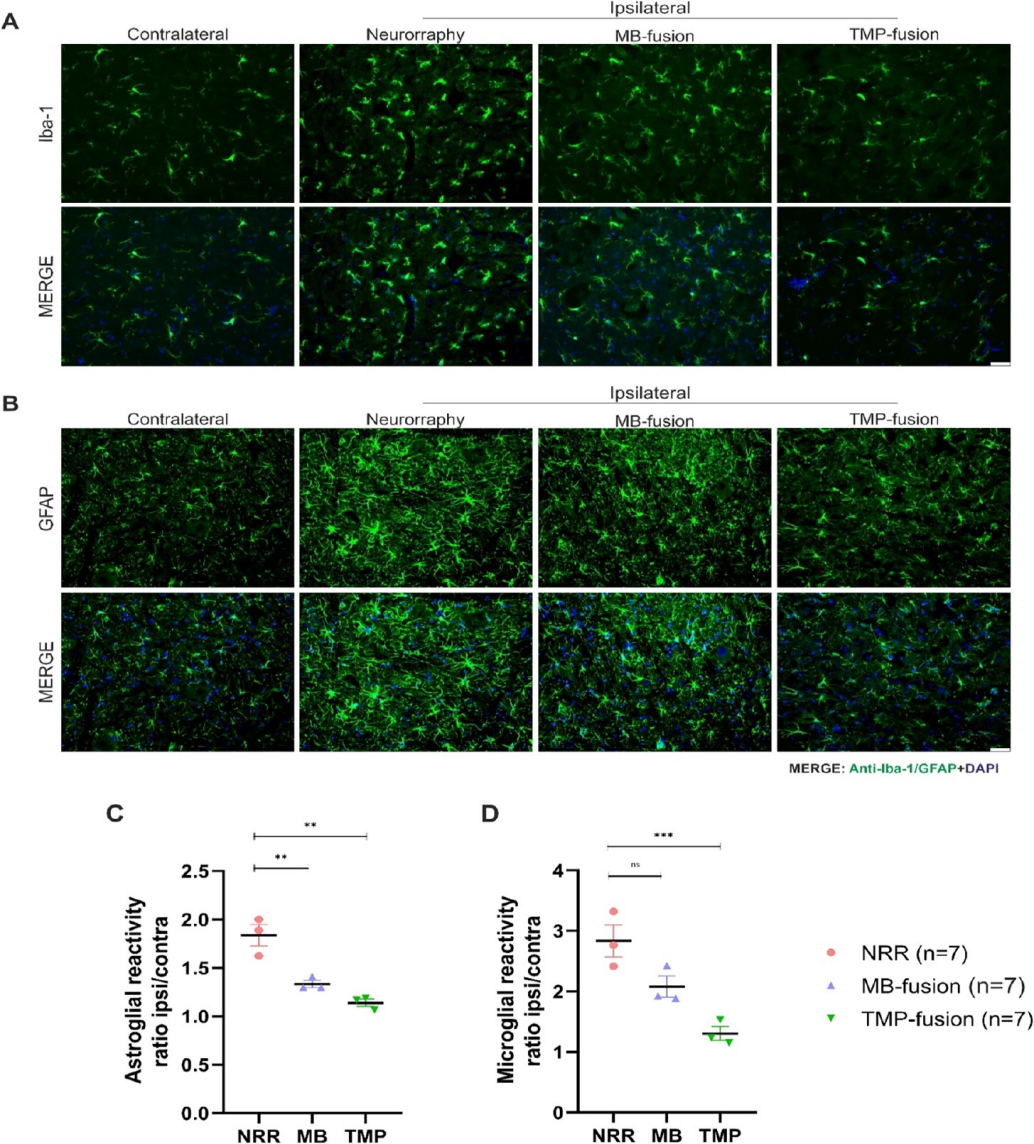
(A) Representative images of immunolabeled microglia (Iba-1) and (B) astrocytes (GFAP) in lamina IX of Rexed (lumbar spinal cord, L4-L6) 8 weeks after treatment following nerve transection. A significant reduction in microglial and astroglial reactivity was observed in the TMP-fusion group. (C) Quantification of astroglial reactivity. (D) Quantification of microglial reactivity. One-way ANOVA followed by Tukey’s post hoc test (*p* < 0.05 (*); *p* < 0.01­(**); *p* < 0.001 (***); *p* < 0.0001 (****)). Scale bar: 50 μm.

Quantitative analysis revealed that the Tempol treatment significantly reduced microglial reactivity from 2.87 ± 0.13 (NRR group) to 1.30 ± 0.11 (*p* = 0.0001), whereas the MB- fusion treatment resulted in a reduction to 2.10 ± 0.16, though this difference was not statistically significant compared to the control (*p* = 0.0017) (Figure 6C). Similarly, astrocytic reactivity was significantly reduced with the TMP-fusion treatment, decreasing from 1.75 ± 0.07 (NRR group) to 1.16 ± 0.03 (*p* = 0.0001). The MB-fusion treatment also showed a significant reduction compared to the NRR (1.36 ± 0.04, *p* = 0.0003) (Figure 6D). In summary, these results suggest that Tempol has immunomodulatory properties, significantly reducing glial reactivity compared to the other treatments.

Tempol also significantly reduced microglial and astrocytic reactivity in lumbar spinal cord segments. Although glial cells normally support motoneurons, injury induces a reactive phenotype that promotes neuroinflammation and glial scarring, compromising synaptic integrity and neuronal survival.^95,97,98^ Tempol’s ability to suppress this activation—likely via downregulation of proinflammatory cytokines such as IL-1β, TNF-α, and IL-6—contributes to a more permissive regenerative environment.^99^

Overall, our findings show that Tempol’s immunomodulatory and neuroprotective effects preserve synaptic architecture, enhance motoneuron survival, and maintain spinal cord circuitry. Among the PEG-fusion protocols, Tempol-fusion achieved the most favorable functional, electrophysiological, and morphological outcomes, likely due to its superior antioxidant and anti-inflammatory properties compared to MB. Further studies should explore Tempol’s molecular mechanisms and the use of adjunctive strategies—like nerve-supporting devices—to optimize axonal fusion and stability.^2,40,73^

#### Tempol Limits Myelin Degeneration Events

Morphological and quantitative analyses of peripheral nerves, two weeks after NRR, MB- or TMP-fusion, revealed clear differences in the degree of myelin degeneration across groups. Toluidine blue staining of semithin sections (Figure 7A, top) shows a more pronounced pattern of axonal and myelin disorganization in the NRR group, whereas MB-fusion and, particularly, TMP-fusion exhibit preserved fibers and reduced accumulation of myelin debris. The Artificial Intelligence (AI) assisted semiautomated segmentation of degenerating myelin profiles (Figure 7A, bottom) reinforces the above-mentioned pathological changes. The analysis of event density (Figure 7B), quantitatively demonstrated that the NRR group maintained the highest density of degenerating profiles per μm^2^, while MB-fusion and TMP-fusion displayed similar profiles, with the latter showing a statistically significant difference relative to NRR.

**Figure 7:**
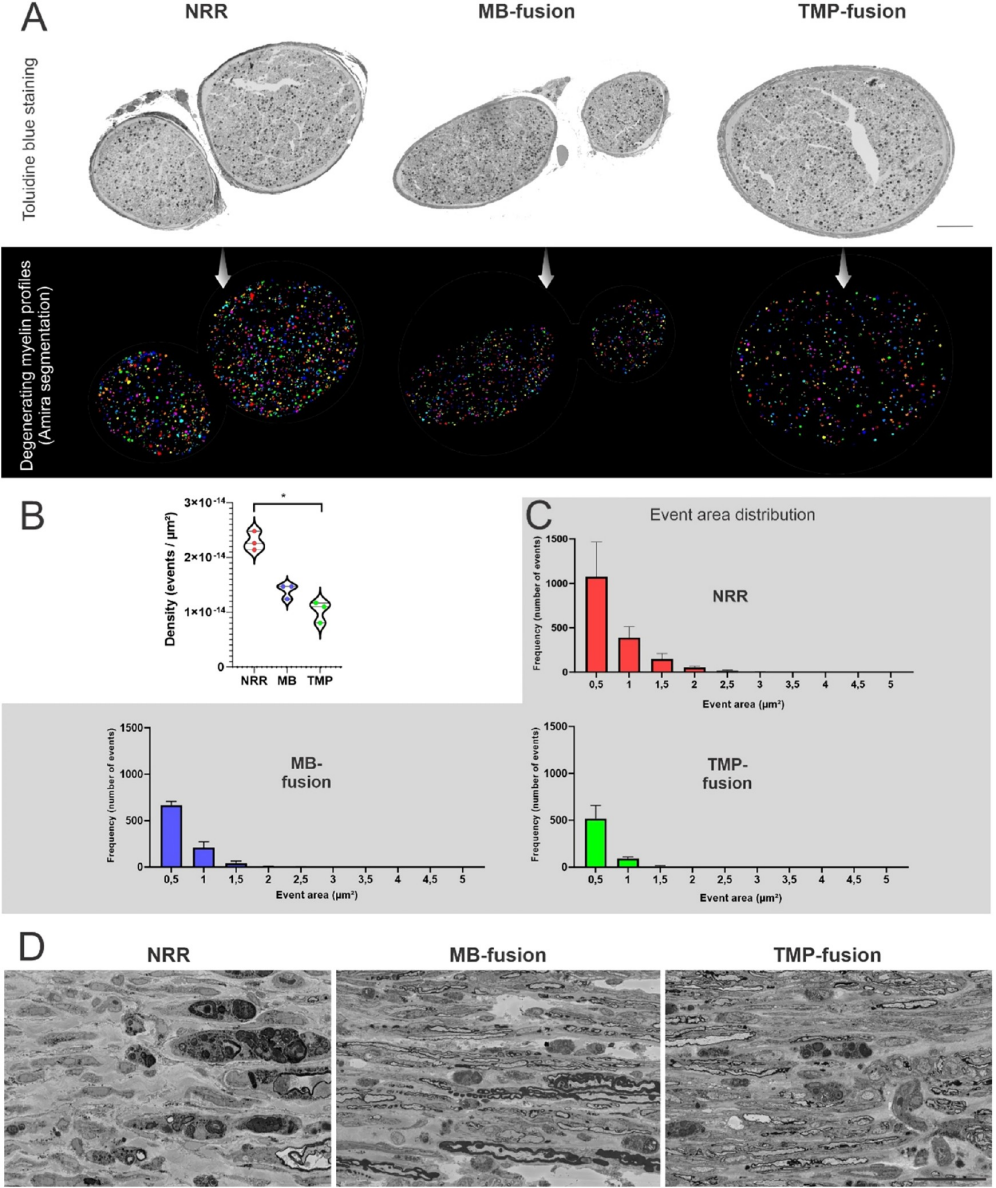
Quantification and ultrastructural analysis of degenerating myelin profiles two weeks after injury. (A) Semithin transverse sections of peripheral nerves stained with toluidine blue in the NRR, MB-fusion, and TMP-fusion groups, accompanied by the segmentation of degenerating myelin profiles. Scale bar: 200 μm. (B) Density of degenerating events (events/μm^2^) across the experimental groups. (C) Distribution of the areas of degenerating events in the three groups. (D) Representative electron microscopy images illustrating the ultrastructural presence of myelinated axons after MB- and TMP-fusion protocols. Scale bar: 40 μm.

The distribution of event areas (Figure 7C) reinforces that following NRR, there was a clear predominance of small-area events (0.005–0.01 μm^2^), accompanied by a tail of larger events, suggesting active degeneration. In contrast, both MB-fusion and TMP-fusion showed a reduction in the overall frequency of events.

Electron microscopy evaluation (Figure 7D) provided ultrastructural support for the quantitative analyses. In NRR, myelin lamellae are disorganized, with evident vacuolization and fragmentation, corroborating the elevated number of degenerating events detected. In MB-fusion, although such signs were still present, they appeared less abundant and more localized; similarly, in TMP-fusion, myelin architecture was slightly more preserved compared to NRR.

Taken together, these findings suggest that mechanisms associated with axonal membrane fusion may confer structural protection to myelin or accelerate the clearance of degenerating profiles, contributing to a more preserved morphology and functional state of the nerve. PEG-fusion, particularly with Tempol, limited early myelin breakdown, aligning with the superior electrophysiological recovery observed in comparison to NRR.

## Limitations

This study presents some limitations that need to be discussed. First, although the rat sciatic nerve transection model is widely used in peripheral nerve research, anatomical and physiological differences limit direct extrapolation to humans. Second, the follow-up period was restricted to 8 weeks; therefore, longer-term studies are required to determine the durability and stability of TEMPOL–PEG-fusion outcomes. Third, although we evaluated functional, electrophysiological, and histological parameters, the specific molecular mechanisms underlying TEMPOL’s neuroprotective and anti-inflammatory effects remain to be elucidated. Finally, natural locomotion may mechanically disrupt fused axons over time, potentially compromising repair stability. Future studies should consider incorporating nerve guidance conduits or stabilization strategies to protect fused axons and improve long-term outcomes.

## Conclusion

Our findings demonstrate that PEG-fusion significantly improved early and long-term recovery after sciatic nerve transection, and its effectiveness was further enhanced by antioxidant treatment. Among all groups, Tempol-PEG-fusion produced the best outcomes, showing faster CMAP restoration, shorter latencies, higher amplitudes, and superior motor recovery. Structural and ultrastructural analyses demonstrated that Tempol most effectively reduced myelin degeneration and preserved axonal organization, aligning with its stronger electrophysiological performance. Overall, Tempol enhances PEG-fusion by limiting oxidative damage, stabilizing axonal membranes, and reducing degenerative changes, making it the most promising approach for improving functional and structural recovery after peripheral nerve injury.

## Experimental Section

### Animals and Experimental Treatments

Seven-week-old female Lewis rats (170–200 g) were obtained from the Multidisciplinary Center for Biological Investigation (CEMIB/UNICAMP) for this experiment. The animals were housed in a certified animal care facility under controlled temperature conditions, with food and water available ad libitum, and maintained on a standard light-dark cycle (lights on: 7:00 a.m. to 7:00 p.m.). Prior to the experiments, all animals underwent a one-week habituation to the housing conditions. This study was approved by the Institutional Committee for Ethics in Animal Experimentation (Committee for Ethics in Animal Use – Institute of Biology – CEUA/IB/UNICAMP, CEUA n◦ 5875–1/2021) and all procedures were performed in compliance with the guidelines established by the Brazilian College for Animal Experimentation.

The study design comprises four experimental groups: NRR (*n* = 7), Fusion–MB (*n* = 7), Fusion–TEMPOL (*n* = 7), and a sham-operated group (*n* = 3) - 8-week survival time. In addition, a separate cohort of animals was used specifically for the electron microscopy protocol, consisting of NRR (*n* = 3), MB–fusion (*n* = 3), and TEMPOL – fusion (*n* = 3) - 2-week survival time. These samples were used for the quantitative assessment of Wallerian degeneration in distal transverse sections and for qualitative 2D reconstruction of the fusion site using serial block-face (SBF) imaging in longitudinal sections. All these groups underwent electrophysiological, functional, and histological assessments as appropriate for each condition. Table 1 summarizes the experimental groups.

**Table 1:** Treatment Groups and Experimental Procedures^a^

**group**	**treatment description**	**procedure**	***N***
**Sham**	surgical exposure without injury	ENMG, IF, Catwalk	3
**NRR**	neurorraphy (gold standard, negative control)	ENMG, IF, Catwalk	7
		electron microscopy protocol, SBF	3
**MB-fusion**	PEG-fusion + methylene blue	ENMG, IF, Catwalk	7
		electron microscopy protocol, SBF	3
**TMP-fusion**	PEG-fusion + tempol	ENMG, IF, Catwalk	7
		electron microscopy protocol, SBF	3

a This table summarizes all treatment groups, providing an overview of the experimental techniques. Abbreviations: ENMG: electroneuromyography; IF: immunofluorescence; Catwalk: Walking track test; Serial block-face: SBF.

### Surgical Procedure for PNI

Rats were anesthetized with ketamine (10 mg/kg, i.p.) and xylazine (100 mg/kg, i.p.), and maintained with 1% inhaled isoflurane/oxygen mixture. The surgical and PEG-fusion protocols followed established methods (Ghergherehchi et al. and Bittner et al.).^14,26^

A lateral incision was made on the right hindlimb to expose the sciatic nerve. The fascia was opened, and the biceps femoris muscle was bluntly separated along its natural plane. The nerve was carefully freed from the surrounding connective tissue and kept hydrated with calcium-supplemented saline (see Table 2 and Figure 8). In the NRR group, an epineurial suture was performed with careful alignment of the nerve stumps. In the fusion groups, the nerve repair followed the fusion protocol described below.

**Figure 8:**
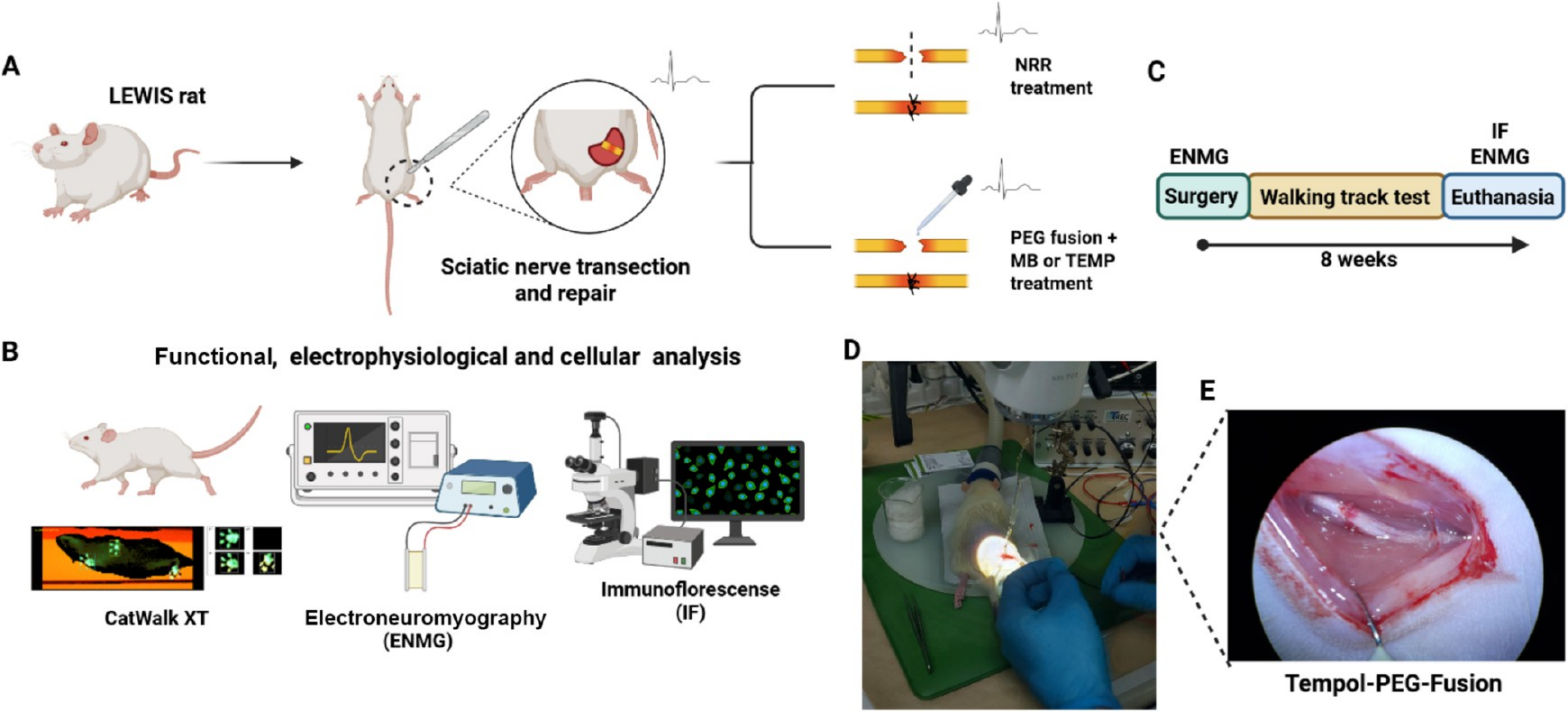
Overview of Experimental Procedure. (A) Adult female Lewis rats underwent unilateral sciatic nerve transection, followed by immediate treatment with one of three protocols: end-to-end neurorrhaphy (NRR), Methylene Blue PEG-fusion (MB-PEG-fusion), or Tempol PEG-fusion (TMP-PEG-fusion). (B) Schematic timeline summarizing the key steps of the experimental protocol, including surgical procedures, assessments, and end point analyses. (C) Electroneuromyography (ENMG) was performed at three time points: baseline (prelesion), immediately after fusion, and at 8 weeks postsurgery. Functional recovery was evaluated weekly over 8 weeks using the walking track test. At the 8-week end point, immunohistochemical analysis was also conducted on sciatic nerve sections (S100 and neurofilament) and spinal cord tissue (synaptophysin, Iba1, and GFAP) (D) Intraoperative image captured during the surgical procedure. (E) Sciatic nerve appearance immediately following Tempol-PEG-fusion. Created with BioRender.com.

**Table 2:** Saline Solutions used in the PEG-Fusion Protocol, as Described by Ghergherehchi et al. (2021)^a^

**solution**	**composition** (mg/100 mL)	**note**
**Normosol-R**	526 mg NaCl	used as hypotonic calcium free solution
	222 mg C2H3NaO2	
	502 mg NaC6H11O7	
	37 mg KCl	
	30 mg MgCl2	
**Lactated Ringer’s**	600 mg NaCl	used as isotonic calcium containing solution
	310 mg C_3_H_5_NaO_3_	
	30 mg KCl	
	20 mg CaCl_2_	

a Normosol-R was employed as a hypotonic, calcium-free solution, while Lactated Ringer’s was used as an isotonic, calcium-containing solution.

### PEG-Fusion Protocol

After sciatic nerve transection, the PEG-fusion procedure was applied in five steps (Table 2 and Figure 9):

1. **Ca** ^**2+**^ **-Free Hypotonic Saline** (Normosol-R) irrigated the lesion for 2 min.
2. An **antioxidant solution** (Methylene Blue or Tempol) was applied for 2 min.
3. **Neurorrhaphy** was performed using 10–0 nylon sutures.
4. A **50% PEG solution** was applied for 2 min to promote axonal membrane fusion.
5. The site was irrigated with **calcium-containing isotonic saline** for 2 min.

**Figure 9:**
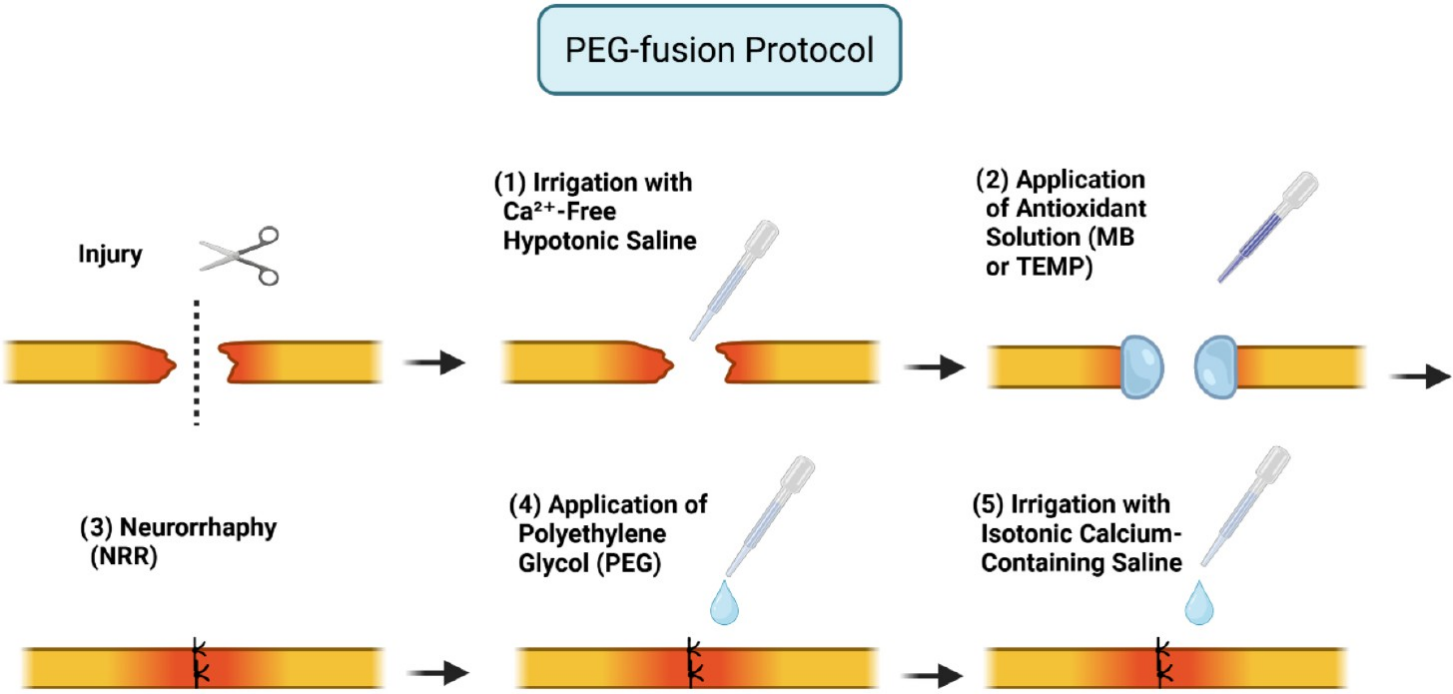
PEG-Fusion Protocol. This figure illustrates the sequential steps of the PEG axonal fusion protocol used in this study. The protocol involved: (1) after injury, irrigation with Ca^2+^-free hypotonic saline, (2) application of an antioxidant solution (Methylene Blue or Tempol), (3) neurorrhaphy, (4) application of a polyethylene glycol (PEG) solution to promote axonal membrane fusion, and (5) final irrigation with isotonic calcium-containing saline. Created with BioRender.com.

Following closure, tramadol (5 mg/kg) was administered subcutaneously for 3 days. Animals were monitored daily for signs of pain, infection, or distress.

#### Sham-Operated Group

A sham-operated group (n = 3) was included to control for nonspecific effects of surgical exposure. In these animals, the sciatic nerve was exposed following the same surgical approach used for the experimental groups, but no nerve injury was produced. The muscle and skin were closed using the same suture procedures of the other experimental groups.

Sham animals were monitored using the walking track test for 4 weeks to assess potential functional alterations associated with the surgical procedure. After the 4-week monitoring period, animals were euthanized and transcardially perfused with 0.1 M phosphate-buffered saline (PBS) followed by 4% paraformaldehyde (PFA). Sciatic nerves were then collected for immunofluorescence staining using antineurofilament antibody to evaluate axonal morphology.

### Electroneuromyography (ENMG)

ENMG was conducted at baseline, immediately after treatment, and 8 weeks postsurgery to evaluate sciatic nerve function via compound muscle action potentials (CMAPs).

The sciatic nerve was stimulated using bipolar electrodes; responses were recorded from the cranial tibial muscle, amplified (100×), and digitized. Stimulation was applied with bipolar stainless-steel electrodes placed on the exposed sciatic nerve proximal to the injury site. Single bipolar pulses (100 μs ± 1600 μV) were delivered using a multichannel stimulator. Complete transection was confirmed by the absence of CMAPs postinjury. Contralateral nerves served as internal controls.

Measured parameters included latency (ms), total and positive durations (ms), and amplitudes (mV). Data were analyzed using image analysis software (Figure 10).

**Figure 10:**
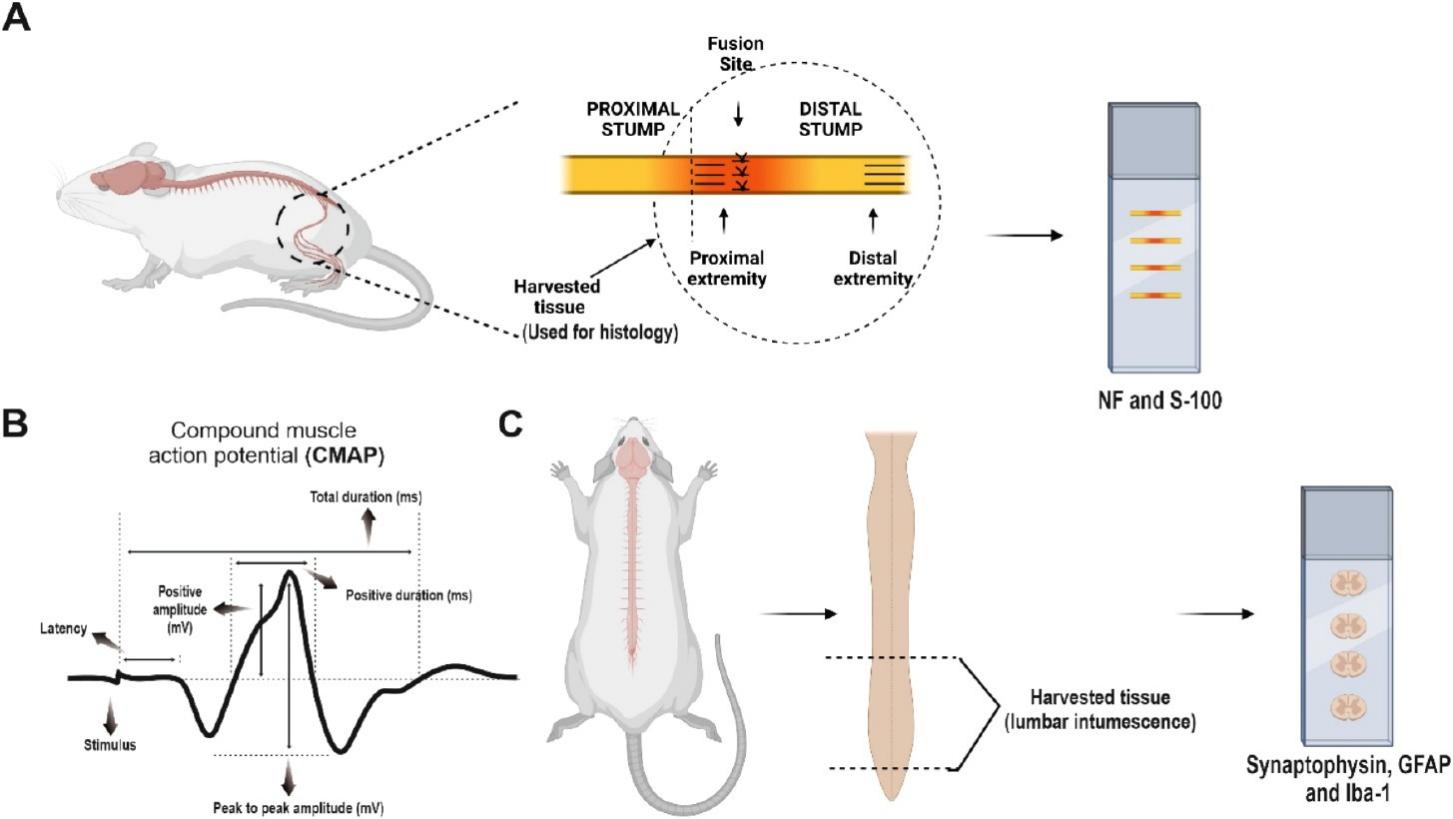
Histological analysis and Electroneuromyography (ENMG). (A) The distal stump of the sciatic nerve was collected for histological analysis, focusing on structural and cellular evaluation of the injury site using S100 and neurofilament immunolabeling. (B) Representative ENMG trace illustrating the compound muscle action potential (CMAP) with parameters measured: latency, total duration, and total amplitude. Positive duration and positive amplitude were also analyzed, as these are clinically relevant measurements. (C) The lumbar intumescence of the spinal cord was also harvested for histological analysis, specifically to evaluate synaptic and glial responses using synaptophysin, GFAP, and Iba-1 markers. Created with BioRender.com.

### Animal Euthanasia and Tissue Preparation

At 8 weeks postinjury, animals were anesthetized with xylazine (100 mg/kg)/ ketamine (10 mg/kg, i.p.) and maintained under 1% isoflurane. After confirming deep anesthesia, CMAPs were recorded, followed by transcardial perfusion with PBS and 4% paraformaldehyde in 0.1 M phosphate buffer (pH 7.4).

For immunofluorescence, the lumbar spinal cord and both sciatic nerves were postfixed (12 h, 4 °C). The distal injured nerve segment was reserved for histology (Figure 10). Tissues were cryoprotected in graded sucrose, embedded in O.C.T., frozen in chilled *n*-hexane (−35 °C), and stored at −22 °C. Twelve-micrometer transverse (spinal cord) and longitudinal (nerve) sections were collected on gelatin-coated slides for further analysis. Immunofluorescence staining with antineurofilament and S-100 was used to assess axonal integrity and Schwann cells, respectively. Images were taken at the proximal (PE) and distal (DE) extremities of the distal stump, with three images per extremity for quantitative analysis (Figure 10).

### Immunofluorescence

Immunofluorescence was performed to assess the nerve microenvironment, synaptic integrity, and glial activation. After blocking with 3% BSA in 0.01 M PB (1h), tissue sections were incubated overnight (4 °C) with primary antibodies diluted in 1% BSA and 0.2% Triton X-100 in PB (Table 3), followed by AlexaFluor 488-conjugated secondary antibodies (1:500, 45 min, room temperature).

**Table 3:** Primary Antibodies for Immunofluorescence^a^

**primary antibodies**	**target**	**supplier**	**host**	**cat**. **number**	**dilution**
Anti-S100	Schwann cells	Abcam	rabbit	Ab868	1/1500
Anti-Neurofilament	Axons	Millipore	rabbit	AB1989	1/2000
Anti-Synaptophysin	Synaptic vesicles	Novus Biologicals	rabbit	NBP2-25170	1/1000
Anti-GFAP	Astrocytes	Abcam	rabbit	Ab7260	1/750
Anti-Iba-1	Microglia	Wako	rabbit	019-19741	1/750

a Table summarizes primary antibodies used for histological analysis, detailing their target, supplier, host species, product code, and optimal working dilutions.

Sections were mounted in glycerol/PB (3:1) and imaged using a fluorescence microscope. Six fields per animal (three proximal, three distal) were analyzed. Quantification of fluorescence intensity was performed with ImageJ, following Oliveira et al.,^100^ and expressed as mean ± SEM.

### CatWalk: Motor Evaluation of Functional Recovery

Motor recovery was evaluated twice weekly for 8 weeks using the CatWalk XT system.^101^ Baseline gait was recorded presurgery. Rats traversed an illuminated walkway while paw placement and pressure were captured by a high-speed camera (Fujinon DF6H-1B, 8.5 mm lens).

Three parameters were analyzed:

1. **Peroneal Functional Index (PFI):** Assessed motor recovery based on paw print dimensions, per Bain et al.^102^
2. **Regularity Index:** Measured interlimb coordination from step sequence patterns.
3. **Base of Support:** Calculated hind paw spacing to evaluate balance.

Together, these parameters provided a comprehensive analysis of locomotor function following sciatic nerve injury.

### Electron Microscopy

#### Euthanasia and Dissection of the Distal Nerve Segment

To complement the semiquantitative imaging data and provide a higher-resolution assessment of nerve structure, we performed an electron microscopy protocol using sciatic nerve samples from each experimental group: NRR, MB–Fusion, and TEMPOL–Fusion (*n* = 3 per group). Two weeks after surgery, animals were transcardially perfused with PBS followed by Karnovsky’s fixative (2% glutaraldehyde +1% paraformaldehyde in 0.1 M phosphate buffer). A 3–4 mm segment distal to the repair site (neurorrhaphy with or without fusion) was excised and immersed in fresh Karnovsky’s solution.

#### Heavy-Metal Staining for Electron Microscopy (OTO – Thiocarbohydrazide–Osmium Cycling)

Samples were processed using the heavy en bloc staining protocol described by Hua et al. (2015), optimized for volumetric acquisition by SBF-SEM. Briefly, after initial fixation, tissues underwent postfixation in 2% osmium tetroxide (OsO_4_) reduced with 2.5–3% potassium ferrocyanide. Subsequent metal enhancement was achieved using a 1% thiocarbohydrazide (TCH) cycle followed by 2% OsO_4_, resulting in stratified osmium deposition. Samples were then contrasted in 1% uranyl acetate at 4 °C (overnight) and subsequently impregnated with lead aspartate (pH 5.0–5.5). After metalization, tissues were dehydrated through a graded ethanol series, transitioned to anhydrous acetone, and infiltrated with epoxy resin (25, 50, 75, and 100%), followed by polymerization at 60 °C.

#### Semithin Sections and Quantitative Analysis of Wallerian Degeneration

Semithin transverse sections (0.5 μm thick) from distal nerve segments, prepared with the electron microscopy fixation protocol, were stained with toluidine blue and imaged using an Axioscan 7 slide scanner (Carl Zeiss Microscopy, Germany). Quantitative assessment of Wallerian degeneration was performed by counting degenerating axons (myelin debris) and normalizing these values to the cross-sectional area of the sciatic nerve in each sample across the NRR, MB–fusion, and TEMPOL–fusion groups.

##### Segmentation and Quantitative Analysis

The segmentation pipeline was performed in Amira 3D 2024.1 (Thermo Fisher Scientific) and followed a unified workflow composed of three sequential stages. Initially, the raw data sets were converted into a scalar format compatible with the segmentation modules (*Convert Image Type*), generating the base image used throughout the entire process. This image then underwent AI-assisted segmentation (*Assisted Segmentation*), followed by semiautomatic refinement through the removal of small, disconnected islands (*removeIslands*). To enable marker construction for the watershed algorithm, an Euclidean distance map (*Chamfer Distance Map*) was calculated, and regional maxima were identified using the *H-Maxima* module, which provided potential object centers. A preliminary label field was subsequently generated using the Labeling module, forming the initial reference for segmentation.

Following this preparatory phase, the intensity image was inverted using the *NOT* module, producing the negative topography necessary for the watershed flooding procedure. The markers derived from the H-Maxima module, together with the distance map and the inverted intensity image, were integrated into the *Marker-Based Watershed* module, which executed the definitive separation of adjacent structures. The output of this process constituted the final segmentation employed for subsequent quantitative analyses.

Finally, a postprocessing stage was performed to refine the segmented data. The inverted image was combined with the region-of-interest mask using the *AND NOT* Image module, spatially restricting the set of objects to be labeled. The Labeling module was applied again, generating a consolidated label field. This labeled data set was then processed using the *Label Analysis* module, which extracted geometric and spatial parameters for each segmented object, including area, count, and spatial distribution. The accuracy and consistency of the segmentation were qualitatively assessed through visual inspection using the *Ortho Slice* module.

### Serial Block-face Analysis of the Fusion Site

Electron microscopy data sets obtained by Serial Block-Face Scanning Electron Microscopy (SBF-SEM) were acquired using an Apreo 2 VolumeScope operating under high vacuum (Thermo Fisher Scientific). Images were collected with the in-lens T1 detector at an accelerating voltage of 1.78 kV, a beam current of 0.20 nA, and a dwell time between 2.0–5.0 μs, with the working distance maintained at 6.50 mm.

Longitudinal sections of the fusion/suture region were imaged in 2D to assess axonal continuity, alignment, and structural organization at the repair site. The images were aligned and analyzed to compare fusion morphology across the three experimental groups, enabling the identification of fused axons, the degree of tissue integration, and morphological differences among treatments.

### Statistical Analysis

Statistical analyses were performed using GraphPad Prism 7.0.5. ENMG data were analyzed by one-way ANOVA with Tukey’s post hoc test. Immunofluorescence and CatWalk results were evaluated using two-way ANOVA with Bonferroni correction. Density measurements were analyzed using the Kruskal–Wallis test followed by Dunn’s multiple comparisons test. Data are presented as mean ± SEM. Significance was set at *p < 0.05; **p < 0.01; ***p < 0.001; ****p < 0.0001.

## Supplementary Material



## Notes

Supporting Figure 1: Functional, electrophysiological, and histological characterization of the sham-operated group, including CatWalk gait analysis parameters, representative run images, intraoperative CMAP recordings, and sciatic nerve histology (Sudan Black and neurofilament immunofluorescence). Supporting Figure 2: Electroneuromyographic evaluation of CMAP parameters (latency, duration, and amplitude) recorded before injury, immediately after treatment, and 2 weeks postsurgery in NRR, Fusion–MB, and Fusion–TEMPOL groups, including representative CMAP traces (PDF)

## Abbreviations

TMP: TEMPOL
MB: methylene blue
PEG: polyethylene glycol
NRR: neurorraphy
CMAPs: compound muscular action potentials
PFI: peroneal functional index
PNI: peripheral nerve injury
NMJ: neuromuscular junctions
ROS: reactive oxygen species
ENMG: electroneuromyography
IF: immunofluorescence
