# Supplementary material for: TEMPOL Enhances Polyethylene Glycol Axon Fusion Following Sciatic Nerve Transection in Adult Rats
Source: ACS Chem Neurosci. 2026 Feb 27;17(7):1269–85. doi: 10.1021/acschemneuro.5c00677 (PMC13047541; doi:10.1021/acschemneuro.5c00677)
Supplement: Supplementary file 1 [file cn5c00677_si_001.pdf]

Supporting Information

TEMPOL enhances polyethylene glycol axon fusion following sciatic nerve transection in adult rats

Lynn Ana Flavia Salamanca-Guillén[1], Liana Melo-Thomas[2], Kelly C. S. Roballo[3], André Schwambach Vieira[4], Bruno Henrique de Melo Lima [1], Luciana Politti Cartarozzi[1,5], Alexandre Leite Rodrigues de Oliveira [1,5]*

[1] Lab. of Nerve Regeneration, Department of Structural and Functional Biology, Institute of Biology, University of Campinas, Campinas 13083-907, São Paulo, Brazil.

[2] Exptl. and Physiological Psychology, Philipps-University of Marburg, Marburg 35037, Germany

[3] Edward Via College of Osteopathic Medicine, Blacksburg, VA, USA; Virginia Maryland College of Veterinary Medicine, Virginia Tech, Blacksburg, Virginia 24061, USA.

[4] Department of Biochemistry and Tissue Biology, Institute of Biology, University of Campinas, Campinas 13083-862, São Paulo, Brazil.

[5] Center for Gender-specific Biology and Medicine (CGBM), Campinas 13083-862, São Paulo, Brazil

Supplementary Figure S1. Functional, Electrophysiological, and Histological Characterization of the Sham Group

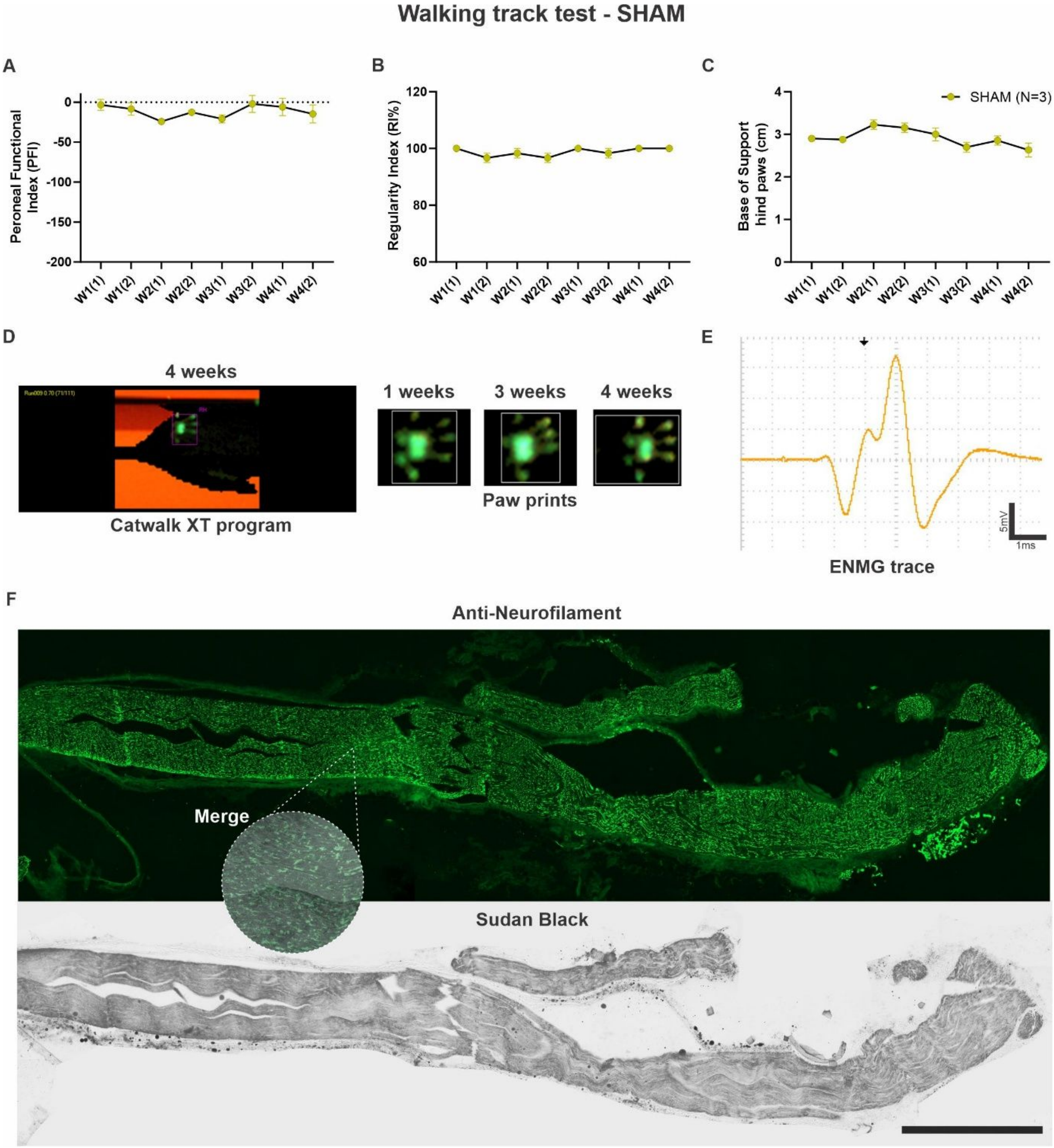

Supplementary Figure S1. Functional, electrophysiological, and histological characterization of the sham-operated group. (A) Peroneal Functional Index (PFI), (B) Regularity Index (RI%), and (C) Base of Support (cm) obtained with the CatWalk gait analysis system over four postoperative weeks. No decrease in motor function was detected, confirming that sciatic nerve surgical exposure alone does not impair locomotion and supporting the idea that the surgical manipulation itself did not alter nerve function. (D) Representative CatWalk run image from week 4 showing normal and symmetric gait patterns together with footprint patterns from weeks 1, 3, and 4, demonstrating stable paw placement and the absence of compensatory gait changes over time, consistent with an intact sciatic nerve. (E) Electroneuromyography (ENMG) recorded intraoperatively, showing a compound muscle action potential (CMAP) with amplitude and duration comparable to normal values (scale: 5 mV, 1 ms), further indicating preserved electrophysiological integrity. (F) Histological analysis of the sciatic nerve using anti-neurofilament immunofluorescence and Sudan Black staining, and merged images. The sham-operated nerve displays continuous axonal morphology, intact myelin, and no evidence of Wallerian degeneration or inflammatory changes. Scale bar: 1000 μm.

Supplementary Figure S2. Electrophysiological assessment of experimental groups used for electron microscopy.

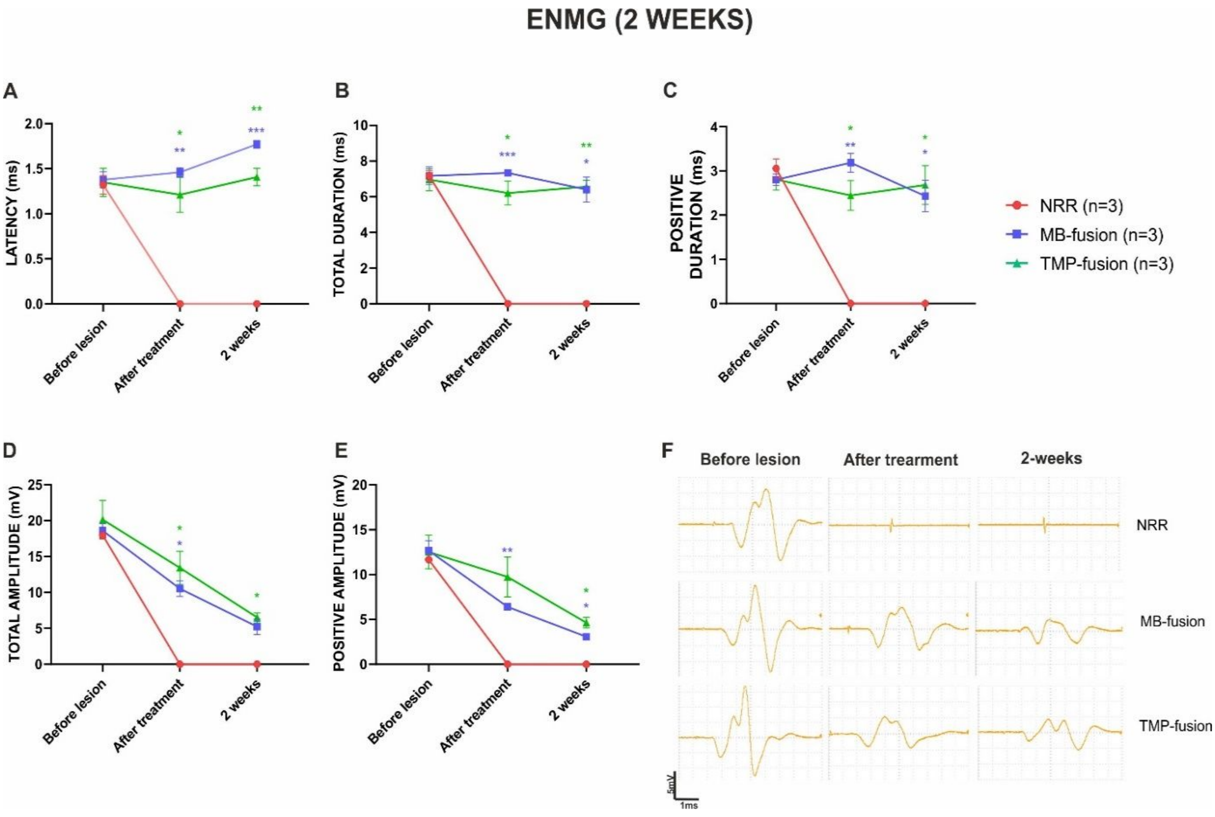

Supplementary Figure S2. Electroneuromyographic (ENMG) evaluation of animals used for the electron microscopy protocol. (A) Latency (ms), (B) total duration (ms), (C) positive duration (ms), (D) total amplitude (mV), and (E) positive amplitude (mV) of the compound muscle action potentials (CMAPs) recorded at three time points: before injury, immediately after treatment, and at two weeks post-surgery. Data correspond to the NRR, Fusion–MB, and Fusion–TEMPOL groups used for the electron microscopy

experiments (n = 3 per group). After two weeks, no detectable CMAPs were observed in the NRR group, whereas both fusion treatments (MB and TEMPOL) showed recovery of electrical conductivity, consistent with improved axonal continuity. (F) Representative CMAP traces illustrating the electrophysiological differences across time points and treatment groups. Statistical analysis was performed using two-way ANOVA followed by Dunnett's multiple comparison test. Significance levels: $p < 0.01$ (*), $p < 0.005$ (**), $p < 0.001$ (***).
